# Green Afterglow of Undoped SrAl_2_O_4_

**DOI:** 10.3390/nano11092331

**Published:** 2021-09-09

**Authors:** Bao-Gai Zhai, Yuan-Ming Huang

**Affiliations:** School of Microelectronics and Control Engineering, Changzhou University, Changzhou 213164, China; bgzhai@cczu.edu.cn

**Keywords:** strontium aluminate, afterglow, luminescence center of afterglow, oxygen vacancy, thermoluminescence

## Abstract

Undoped SrAl_2_O_4_ nanocrystals were obtained via solution combustion using urea as fuel. The afterglow properties of undoped SrAl_2_O_4_ were investigated. Green afterglow from undoped SrAl_2_O_4_ is visible to the human eye when the 325 nm irradiation of a helium–cadmium laser (13 mW) is ceased. The afterglow spectrum of undoped SrAl_2_O_4_ is peaked at about 520 nm. From the peak temperature (321 K) of the broad thermoluminescence glow curve, the trap depth of trap levels in undoped SrAl_2_O_4_ is estimated to be 0.642 eV using Urbach’s formula. Based on first-principles density functional calculations, the bandstructures and densities of states are derived for oxygen-deficient SrAl_2_O_4_ and strontium-deficient SrAl_2_O_4_, respectively. Our results demonstrate that the green afterglow of undoped SrAl_2_O_4_ originates from the midgap states introduced by oxygen and strontium vacancies. The observation of green afterglow from undoped SrAl_2_O_4_ helps in gaining new insight in exploring the afterglow mechanisms of SrAl_2_O_4_-based afterglow materials.

## 1. Introduction

Since the report of green afterglow of Eu^2+^ and Dy^3+^ codoped SrAl_2_O_4_ in 1996, the afterglow properties of Eu^2+^ and an auxiliary trivalent rare-earth ion (Re^3+^) codoped SrAl_2_O_4_ have been intensively investigated due to the advantages of long afterglow time [1,2,3,4,5,6]. After intensive studies over the past 25 years, it is widely accepted that Eu^2+^ is the luminescent center of the afterglow for Eu^2+^ and Re^3+^ codoped SrAl_2_O_4_ phosphors [1,2,7,8,9]. However, this belief has recently been challenged by the following facts: (i) green afterglows peaking at about 520 nm are observed in Dy^3+^ doped SrAl_2_O_4_ and in Tb^3+^ doped SrAl_2_O_4_ [3,4,10,11]; (ii) Finley et al. suggested that the long afterglow of SrAl_2_O_4_:Eu^2+^ stems from native defects in the lattice of SrAl_2_O_4_ on the basis of their density functional studies on charged vacancies in SrAl_2_O_4_ and SrAl_2_O_4_:Eu^2+^ [12]; and (iii) blue and blue–green afterglows are recorded in a variety of undoped materials, among which include HfO_2_ [13], Mg_2_SnO_4_ [14], CaAl_2_O_4_ [15,16], boric oxide [17], and SrSO_4_ [18]. These challenging facts suggest that the native defects in SrAl_2_O_4_, namely the oxygen and strontium vacancies, are likely to be the origin of the afterglow. If so, undoped SrAl_2_O_4_ should exhibit green afterglow since native defects such as oxygen and strontium vacancies are already present in undoped SrAl_2_O_4_ at room temperature. A literature survey indicates that there is no report on the afterglow of undoped SrAl_2_O_4_ so far. The lack of study on the afterglow of undoped SrAl_2_O_4_ makes most of the afterglow mechanisms presented so far vulnerable to serious flaws. Therefore, it is necessary to explore the afterglow features of undoped SrAl_2_O_4_.

In this work, undoped SrAl_2_O_4_ phosphors were obtained via solution combustion in order to explore their afterglow properties. Undoped SrAl_2_O_4_ phosphors are found to exhibit green afterglow when the 325 nm irradiation from a helium–cadmium laser (13 mW) is turned off. The electronic structures of oxygen-deficient SrAl_2_O_4_ and strontium-deficient SrAl_2_O_4_ are derived using density functional theory (DFT) calculations. Our experimental and computational data show that the green afterglow of undoped SrAl_2_O_4_ stems from the oxygen and strontium vacancies in the lattice of SrAl_2_O_4_.

## 2. Materials and Methods

### 2.1. Synthesis of Undoped SrAl_2_O_4_ Phosphors

SrAl_2_O_4_ nanocrystals were prepared by solution combustion technique. All reagents were in analytical grade, details on the chemical suppliers are available elsewhere [3,4,10,11]. Stoichiometric amounts of Sr(NO_3_)_2_·4H_2_O (0.02 mol) and Al(NO_3_)_3_·9H_2_O (0.04 mol) were dissolved in deionized water (100 mL) to form a transparent solution. Urea (0.6 mol) and H_3_BO_3_ (0.002 mol) were added into the solution to work as the fuel of combustion and flux, respectively. The solution had been aged at 40 °C for two weeks before it was transferred into an alumina crucible for combustion. The firing temperature in an air-filled furnace was 740 °C. The resultant solids were ground into fine powders with a pestle in a mortar.

### 2.2. Phase and Morphology of Undoped SrAl_2_O_4_

The X-ray diffractogram (XRD) of the prepared sample was measured with an X-ray diffractometer (model D/max 2500 PC, Rigaku Corporation, Akishima, Japan). The wavelength of the X-ray radiation was 0.15405 nm. The morphology and the energy dispersive X-ray (EDX) spectrum of undoped SrAl_2_O_4_ phosphors were analyzed with the scanning electron microscope (SEM) (model S–4800, Hitachi, Tokyo, Japan). The morphology and the lattice of undoped SrAl_2_O_4_ nanocrystals were determined with a transmission electron microscope (TEM) (model JEOL JEM–2100, Japan Electronics Corp., Akishima, Japan).

### 2.3. Steady-State Photoluminescence (PL) and PL Decay Curves of Undoped SrAl_2_O_4_

The steady-state PL spectrum of undoped SrAl_2_O_4_ was measured with the spectrophotometer (Tianjin Gangdong Ltd., Tianjin, China). A helium–cadmium laser (Kimmon Electric Co. Ltd., Tokyo, Japan) was employed to provide the excitation source. The emission wavelength of the laser was 325 nm, and the excitation power of the laser was 13 mW. The PL decay curves were measured on a picosecond fluorescence lifetime spectrometer (LifeSpec II, Edinburgh Instruments, Edinburgh, UK) utilizing a time correlated single photon counting method. Pulsed light excitation at 320 nm was provided by a picosecond pulsed light emitting diode. At the repetition rate of 10 MHz, the pulse width of the pulsed light was about 860 ps. For each PL decay curve, the pulse duration of the pulsed light source was fixed at 100 ns. Details on the PL decay characterization could be found elsewhere [19,20,21]. Both the steady-state PL and the PL decay curves of undoped SrAl_2_O_4_ were measured at room temperature.

### 2.4. Afterglow Spectrum and Thermoluminescence (TL) Glow Curve of Undoped SrAl_2_O_4_

With the same PL spectrophotometer, the afterglow spectrum of undoped SrAl_2_O_4_ phosphors was recorded immediately after the ultraviolet irradiation from the helium–cadmium laser (325 nm, 13 mW) was blocked off. The TL glow curve of undoped SrAl_2_O_4_ was measured on a TL meter constructed according to the scheme given by Yamashita et al. [22]. Undoped SrAl_2_O_4_ powders were exposed to the ultraviolet irradiation at 254 nm for 5 min before the TL measurement was started. The TL signals of undoped SrAl_2_O_4_ phosphors were recorded as the phosphors were heated from 283 to 483 K at a rate of 2 K/s.

### 2.5. Electron Paramagnetic Resonance (EPR) Measurement of Undoped SrAl_2_O_4_

The X-band EPR spectrum was measured at room temperature using an X-band EPR spectrometer (JEOL JES–FA200, Japan Electronics Corp., Akishima, Japan) with a 100 kHz magnetic field modulator. The magnetic field was monitored with a gaussmeter, and the magnetic field was swept from 3465 to 3565 G with a sweep width of 100 G. The resonance frequency of its cavity was 9.585 GHz, and the microwave power was 20 mW.

### 2.6. Band Structures and Densities of States of Defect-Rich SrAl_2_O_4_

Density functional calculations of the band structures and the densities of states of defect-rich SrAl_2_O_4_ were performed using the density functional theory (DFT) module (Quantumwise Atomistix ToolKit 11.8 package, Copenhagen, Denmark). The exchange–correlation functional was treated within the generalized gradient approximation (GGA) scheme by the Perdew–Burke–Ernzerhof (PBE) potential [23]. Taken from the Inorganic Crystal Structure Database (ICSD), the lattice parameters of monoclinic SrAl_2_O_4_ (*a* = 0.8447 nm, *b* = 0.8816 nm, *c* = 0.5163 nm, and *β* = 93.42°) were used in the present calculations (ICSD number 26466). The considered electronic configurations were 4s^2^4p^6^5s^2^ for Sr, 2s^2^2p^4^ for O, and 3s^2^3p^1^ for Al. A supercell with dimensions of 1 × 1 × 2 was constructed for defect-free SrAl_2_O_4_. Such a supercell was composed of 8 Sr, 16 Al, and 32 O sites. When one oxygen site was vacant, oxygen-deficient SrAl_2_O_4_ resulted. The resultant SrAl_2_O_4_ was denoted as SrAl_2_O_3.875_ in this work. Similarly, strontium-deficient SrAl_2_O_4_ resulted when one strontium site was removed from the supercell. The resultant SrAl_2_O_4_ was denoted as Sr_0.875_Al_2_O_4_ in this work. Double zeta single polarized basis sets were chosen for each element. The cut-off energy for the plane waves was 75 Hartree. The Monkhorst–Pack scheme k-points grid sampling was set at 5 × 5 × 5 for the Brillouin zone. The Brillouin zone sampling and the kinetic energy cutoff were sufficient to guarantee an excellent convergence for the calculated band structures. Details on the density functional calculations were available elsewhere [15,16,24,25].

## 3. Results and Discussion

### 3.1. XRD Pattern and Morphology of Undoped SrAl_2_O_4_

**Figure 1:**
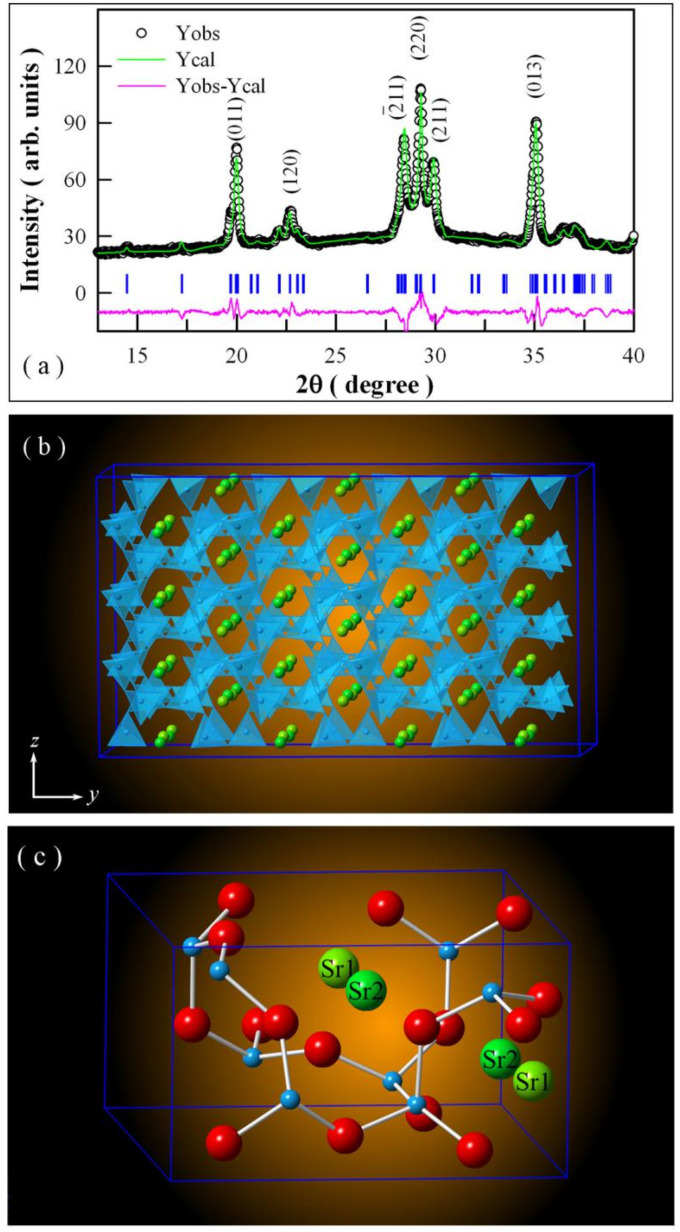
(**a**) XRD pattern of undoped SrAl_2_O_4_ and its Rietveld refinement. Open circles: raw data; solid green curve: Rietveld diffractogram; solid pink curve: residue. (**b**) A supercell of SrAl_2_O_4_ consisting of Sr^2+^ ions situated in the channels of AlO4 tectrahedra. (**c**) Unit cell of SrAl_2_O_4_ showing the two crystallographically different Sr sites.

Figure 1a depicts the XRD pattern of undoped SrAl_2_O_4_ and its Rietveld analysis. In Figure 1a, the raw XRD data are represented by the open circles (in black), and the Rietveld diffractogram of the undoped SrAl_2_O_4_ is represented by the green solid curve. As can be seen in Figure 1a, the XRD profile exhibits distinct peaks at 2θ = 19.95, 22.74, 28.386, 29.275, 29.922, and 35.113°. These peaks match well with the data registered in the Joint Committee on Powder Diffraction Standards (JCPDS) data file (JCPDS card number 34–0379) for monoclinic SrAl_2_O_4_ [3,4,6,10,11]. Hence, according to the data registered in JCPDS card number 34–0379, these peaks can be attributed to the X-ray reflections from crystallographic planes (001), (120), (–211), (220), (211), and (013) of monoclinic SrAl_2_O_4_, respectively. Thus, the monoclinic phase of SrAl_2_O_4_ is confirmed by the XRD pattern in Figure 1a. Using the program FULLPROF Suite 2014, detailed structural information of undoped SrAl_2_O_4_ can be extracted via the Rietveld refinement method, and the lattice parameters derived from the Rietveld refinement are *a* = 0.8427 ± 0.0002 nm, *b* = 0. 8826 ± 0.0002 nm, *c* = 0.5131 ± 0.0001 nm, and *β* = 93.237 ± 0.007°. A comparison of these data with those registered in the ICSD for monoclinic SrAl_2_O_4_ (ICSD #26466) reveals that the unit cell parameters of undoped SrAl_2_O_4_ are close to those of reference SrAl_2_O_4_ (*a* = 0.8447 nm, *b* = 0.8816 nm, *c* = 0.5163 nm, *β* = 93.42°). Monoclinic SrAl_2_O_4_ is known to have the stuffed tridymite structure with the space group P2_1_ and Z = 4. Figure 1b illustrates the schematic view of the monoclinic SrAl_2_O_4_ along the *c*-direction. As can be seen clearly in Figure 1b, monoclinic SrAl_2_O_4_ has a three-dimensional network of corner-sharing AlO4 tetrahedra, and channels are present in the *a*- and *c*-directions. Sr^2+^ ions are located in these channels. Figure 1c displays the unit cell of monoclinic SrAl_2_O_4_. There are two crystallographically different sites for Sr^2+^, which are labeled as Sr1 and Sr2 in Figure 1c. The environments around the two nonequivalent Sr sites differ only by a slight distortion of their square planes [26,27].

**Figure 2:**
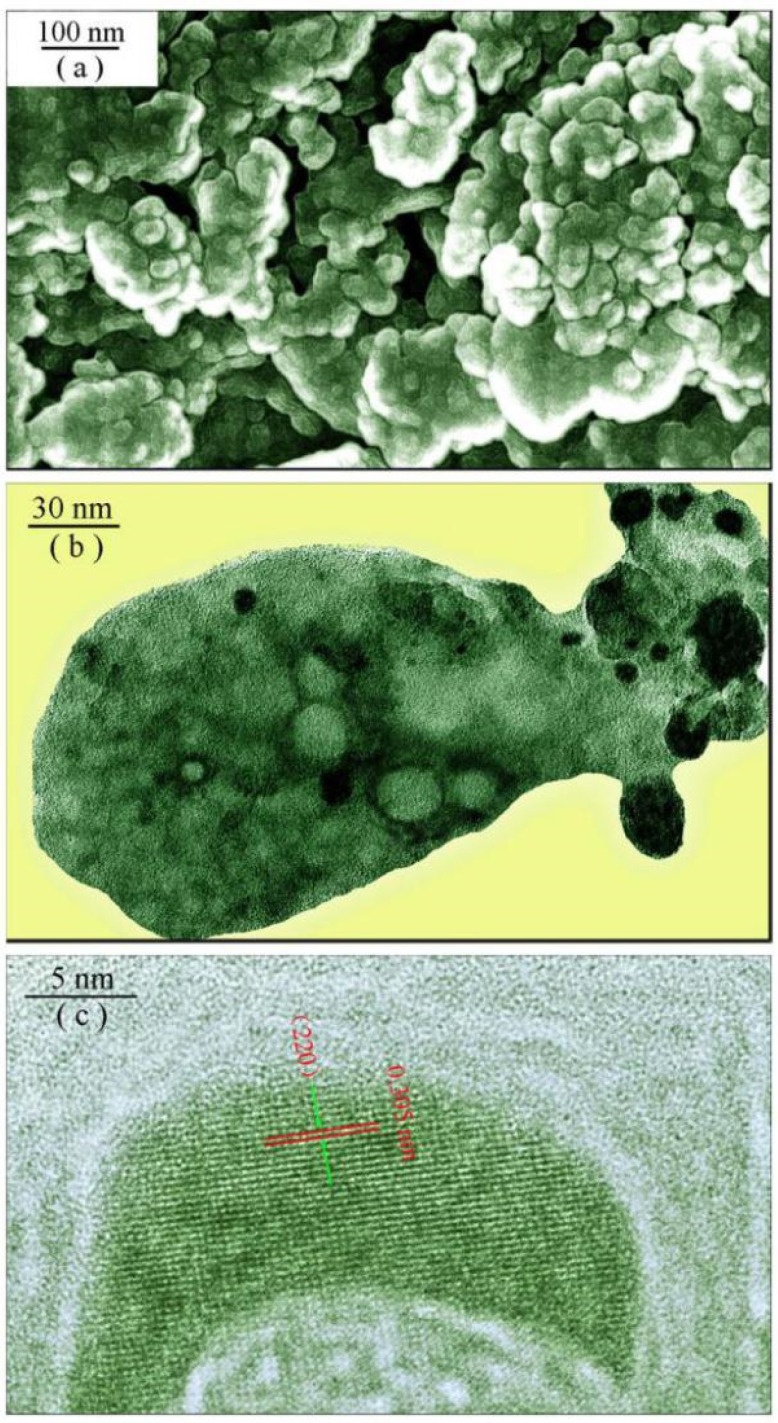
(**a**) SEM micrograph of undoped SrAl_2_O_4_ showing the agglomerates; (**b**) TEM micrograph of undoped SrAl_2_O_4_ showing nanocrystals in an agglomerate; (**c**) High-resolution TEM micrograph of undoped SrAl_2_O_4_ showing the lattice of SrAl_2_O_4_ nanocrystals in an agglomerate.

**Figure 3:**
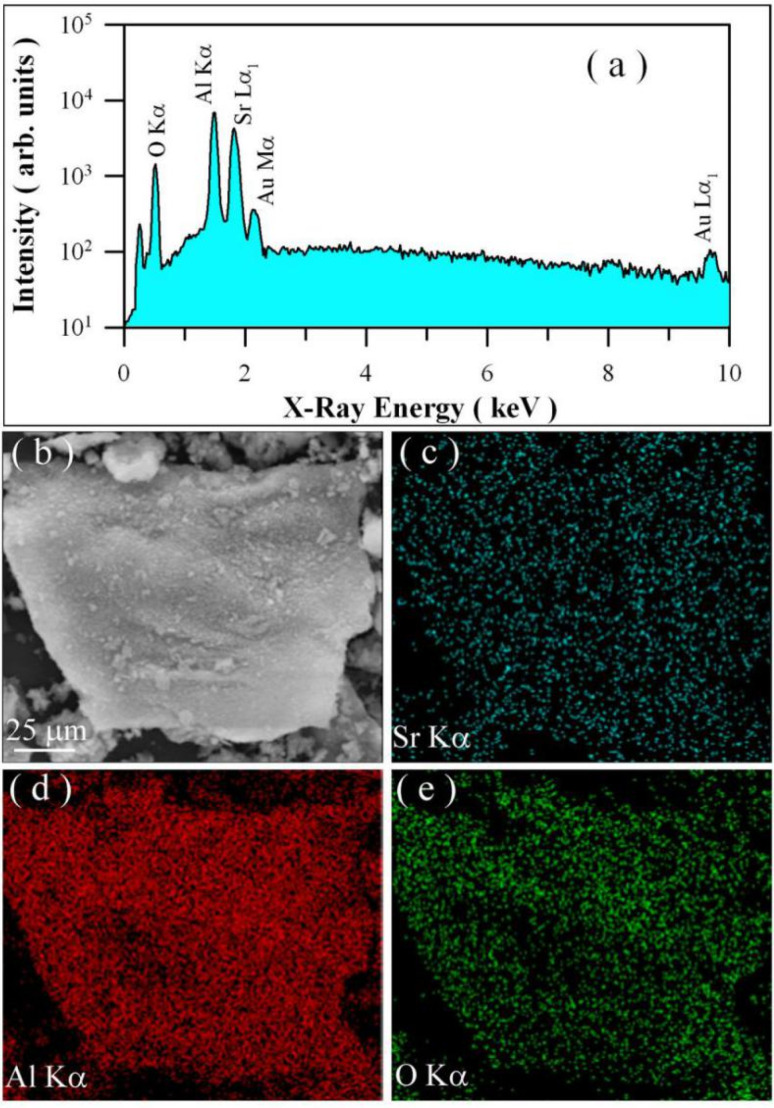
EDX spectrum and elemental mapping of undoped SrAl_2_O_4_ phosphors: (**a**) EDX spectrum; (**b**) electronic image of the selected area for elemental mapping; (**c**) Sr map; (**d**) Al map; and (**e**) O map.

Figure 2 represents the SEM and TEM micrographs of undoped SrAl_2_O_4_. As shown in Figure 2a, the phosphors are in the form of micrometer-sized agglomerates. The largest agglomerate in Figure 2a is about 80 μm in diameter, whilst the smallest one is around 5 μm in diameter. The average particle sizes of the agglomerates are estimated to be 20 μm by means of particle size analyzer. Figure 2b displays the TEM micrograph of an agglomerate of undoped SrAl_2_O_4_. As can be seen in Figure 2b, this agglomerate is about 300 nm in length and 120 nm in width. In particular, some nanocrystals are discernible in this agglomerate, and the sizes of CaAl_2_O_4_ nanocrystals in the agglomerates are less than 30 nm in dimension. Figure 3c shows a high-resolution TEM micrograph of one SrAl_2_O_4_ nanocrystal. As shown in Figure 2c, the spacing between two adjacent planes is 0.305 nm, which agrees reasonably with the distance between two adjacent (220) crystal planes of SrAl_2_O_4_. The clarity of the fringe patterns in the high-resolution TEM micrograph indicates that the synthesized product crystallizes in a single phase. Therefore, an agglomerate of undoped SrAl_2_O_4_ is composed of a large number of SrAl_2_O_4_ nanocrystals [14,15,28].

### 3.2. EDX Spectrum and Element Mapping of Undoped SrAl_2_O_4_

The EDX spectrum of undoped SrAl_2_O_4_ is depicted in Figure 3a. As can be seen in Figure 3a, the characteristic X-ray emissions of O(Kα), Al(Kα), and Sr(Lα_1_) are located at 0.525, 1.486, and 1.806 keV, respectively. Additionally, the X-ray emissions of Au(Mα_1_) and Au(Lα_1_) are identified at 2.122 and 9.713 keV, respectively. As documented previously, the presence of Au element in the specimen is due to the Au sputtering for the convenience of SEM characterization [10,29]. Without considering the element Au, the data in Figure 3a confirm the presence of elements Sr, Al, and O in the synthesized compound. Element mappings are important for displaying element distributions in inorganic materials [30]. Figure 3b shows the electronic image of the selected area of undoped SrAl_2_O_4_, while Figure 3c–e represent the EDX elemental mappings of O, Sr, and Al for the selected area of undoped SrAl_2_O_4_. The mappings demonstrate that the spatial distribution of each element in undoped SrAl_2_O_4_ is uniformly distributed.

It is important to assure that the undoped SrAl_2_O_4_ is not contaminated by a trace of Eu^2+^. Similar to our previous work on undoped CaAl_2_O_4_, we measured the X-ray fluorescence spectrum of undoped SrAl_2_O_4_ under the synchrotron radiation at the energy of 6.99 keV (4W1B endstation, Beijing synchrotron Radiation Facility) [15]. The characteristic X-ray emissions of Eu(Lα_1_) and Eu(Lα_2_) at about 5.84 keV cannot be detected in our undoped SrAl_2_O_4_. Similarly, not even a trace of the Eu^2+^ or Eu^3+^ species could be observed at 6980 eV (for Eu^2+^) or 6988 eV (for Eu^3+^) can be observed in the X-ray absorption spectrum at the L_III_ edge of Eu. Both the X-ray fluorescence and the X-ray absorption analyses demonstrate the absence of Eu^2+^ in undoped SrAl_2_O_4_.

### 3.3. Steady-State PL Spectrum of Undoped SrAl_2_O_4_

**Figure 4:**
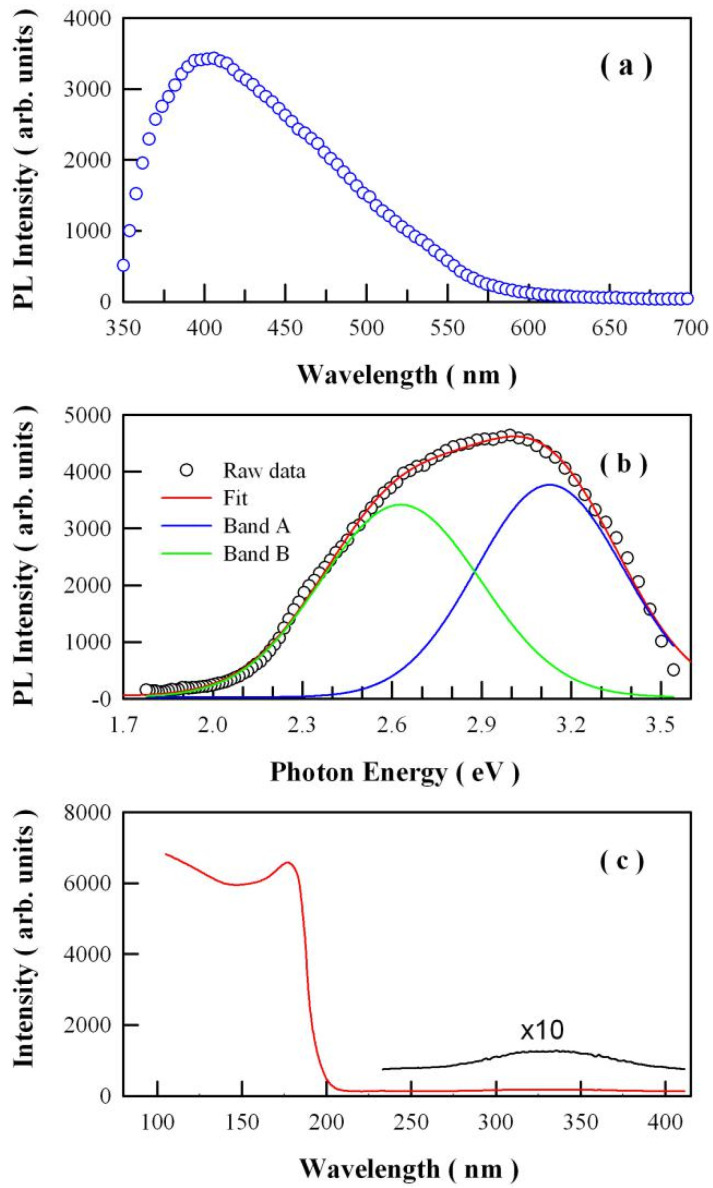
(**a**) Steady-state PL spectrum of undoped SrAl_2_O_4_ in wavelength scale; (**b**) Steady-state PL spectrum of undoped SrAl_2_O_4_ in energy scale; and (**c**) Ultraviolet–vacuum ultraviolet synchrotron radiation excitation spectrum of undoped SrAl_2_O_4_ at room temperature. The emission wavelength is 520 nm.

The steady-state PL spectrum of undoped SrAl_2_O_4_ in wavelength scale is given in Figure 4a. The excitation wavelength is 325 nm. As shown in Figure 4a, the PL spectrum of undoped SrAl_2_O_4_ in wavelength scale is apparently composed of more than one PL band. As mentioned by Mooney et al., the PL spectrum presented in energy scale provides better physical insight [31]. Employing the Jacobian transformation, the PL spectrum in Figure 4a is properly converted into the data in the unit of energy, and the resultant data are shown in Figure 4b. It is found that the PL spectrum in Figure 4b can be deconvoluted into two Gaussian bands. One of the Gaussian bands was centered at 2.63 eV (471.6 nm) while the other was centered at 3.13 eV (396.4 nm). SrAl_2_O_4_ is known as an insulator with a bandgap of 6.52 eV [32,33,34,35]. The incident photon energy of the ultraviolet excitation in this work is only 3.82 eV, which is also limited to trigger the band–edge recombination of SrAl_2_O_4_. Thus, the broad-band emissions in the PL spectrum in Figure 4b can only be originated from the defect related emissions of SrAl_2_O_4_. In SrAl_2_O_4_, oxygen vacancy and strontium vacancy are the two kinds of most common intrinsic defects. Considering the release of a large volume of reducing gases (i.e., CO and NH_3_) during the combustion synthesis, anion vacancy (i.e., oxygen vacancy) is the most favorable defect, whilst cation vacancy is the second most favorable defect in undoped SrAl_2_O_4_. As documented in the literature, oxygen vacancy related emissions are reported for a number of host materials, among which include CaAl_2_O_4_ [17,18,28], SrAl_2_O_4_ [3,4,10,11], BaAl_2_O_4_ [36], SrSO_4_ [18,25], HfO_2_ [13], Mg_2_SnO_4_ [14], ZnWO_4_ [30,37], and ZnMoO_4_ [38]. In the case of SrAl_2_O_4_, Kamada et al. reported that the broad PL spectrum of undoped SrAl_2_O_4_ is comprised of 3 subbands peaking at 250, 360, and 490 nm when excited at 180 nm (6.9 eV) [33], Nazarov et al. reported that the broad PL spectrum of undoped SrAl_2_O_4_ consists of 4 subbands peaking at 250, 375, 450, and 520 nm when excited at 8 eV [39]. These experimental results show that the emission bands in Figure 4b are related to the intrinsic defects in SrAl_2_O_4_. In other words, the oxygen vacancy and strontium vacancy in the lattice of SrAl_2_O_4_ should take the responsibility for the two PL subbands in Figure 4b. Figure 4c illustrates the ultraviolet–vacuum ultraviolet synchrotron radiation excitation spectrum of undoped SrAl_2_O_4_. The emission wavelength is fixed at 520 nm. As can be seen in Figure 4c, intense absorptions take place when the excitation wavelength is shorter than 190 nm, indicating the absorption across the bandgap of SrAl_2_O_4_. In addition to the intense absorption across the bandgap, a weak but broad absorption band can be identified in the range of 250–400 nm. Peaking at about 340 nm, this weak absorption band can be ascribed to the intrinsic defects in SrAl_2_O_4_.

It is important to note here that both oxygen and cation vacancies in oxides can be in several charge states. For example, in the case of oxygen vacancy, they can be doubly positively charged when no electron is trapped (F^2+^ center), singly positively charged when one electron is trapped (F^+^ center), or neutral when two electrons are trapped (F-center). As reported by González et al., both the F^+^ and the F centers in MgO absorb essentially at the same energy of around 5 eV, but their PL peaks are located at 3.2 (F^+^ center) and 2.3 eV (F center), respectively [40]. In the case of oxygen-type vacancies (F^+^ and F centers), photoexcitation leads either to intracenter excitation of the F-type centers or to electron transition from valence band to appropriate unoccupied states within the band gap, and the photoconversions of F^+^ to F centers in MgO and Al_2_O_3_ are addressed [40,41,42]. In our case, the oxygen vacancy in undoped SrAl_2_O_4_ can also have three charge states: doubly positively charged oxygen vacancy when no electron is trapped, positively charged oxygen vacancy when one electron is trapped, and neutral oxygen vacancy when two electrons are trapped. It is understandable that the oxygen vacancies with different charge state exhibit absorption bands in the ultraviolet spectral region and emission bands in the visible spectral region.

Among the oxygen vacancies with different charges states (F, F^+^, and F^2+^ centers), each of them has the possibility of acting as the luminescence center of PL for undoped SrAl_2_O_4_. Upon the continuous irradiation of the ultraviolet laser (325 nm, 13 mW), no obvious change in the PL profile can be observed for undoped SrAl_2_O_4_, suggesting no noticeable photoconversion between the different charged states of oxygen vacancy [40,43]. Note that in undoped SrAl_2_O_4_, charged oxygen vacancies should be thermodynamically stable and energetically feasible. Finley et al. calculated the formation energies of charged oxygen vacancies in undoped SrAl_2_O_4_ as a function of Fermi level at the oxygen-poor limit to mimic the most probable experimental conditions for the synthesis of the SrAl_2_O_4_. Their calculations suggested that: (i) the most energetically favorable vacancy charge state is either a doubly positively charged oxygen vacancy (F^2+^ center) or a neutral oxygen vacancy (F center); (ii) the singly positively charged oxygen vacancy is the least energetically favorable for the anion defects; and (iii) all of the oxygen vacancies with 4 eV above the valence band should have a 2+ charge, whereas the vacancies near the conduction band are likely to be neutral vacancies [12]. Figure 4b shows that each peak energy of the two PL bands in the PL spectrum is less than 3.2 eV, thus the studies performed by Finley et al. suggest that the doubly positively charged oxygen vacancy in SrAl_2_O_4_ is likely responsible for the recorded PL as shown in Figure 4b.

### 3.4. Afterglow Spectrum and Afterglow Decay Profile of Undoped SrAl_2_O_4_

**Figure 5:**
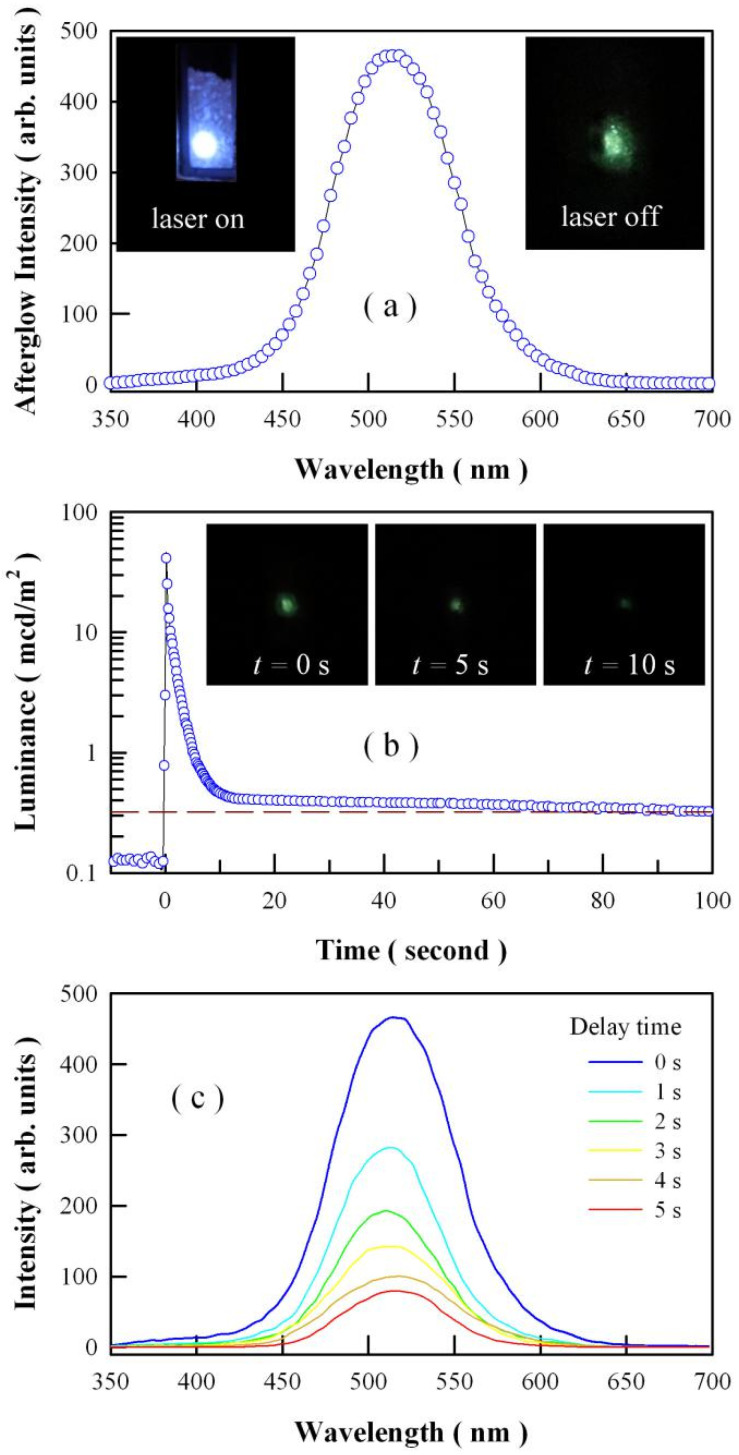
(**a**) Afterglow spectrum of undoped SrAl_2_O_4_. Insets in the top panel: photographs of the undoped SrAl_2_O_4_ when the irradiation of an ultraviolet laser (325 nm, 13 mW) is turned on (left) and off (right). (**b**): Afterglow decay profile of undoped SrAl_2_O_4_, which was taken after exposure to ultraviolet laser of 325 nm for 5 min. Insets in the middle panel: afterglow photographs of the undoped SrAl_2_O_4_ taken at 0, 5, and 10 s after the extinction of the irradiation of the ultraviolet laser. (**c**) Afterglow spectra of undoped SrAl_2_O_4_ measured at different delay times after the extinction of the ultraviolet illumination of the He-Cd laser.

Figure 5a represents the afterglow spectrum of undoped SrAl_2_O_4_. Obviously, this afterglow spectrum is broad in profile with its peak at about 520 nm. It is known that the chromaticity coordinates of the afterglow can be derived through using the data in the afterglow spectrum [43,44]. In the International Commission on Illumination (CIE) 1931 XYZ color space, the chromaticity coordinates of the afterglow are derived to be (0.193, 0.488) for the undoped SrAl_2_O_4_. Hence, the afterglow color of undoped SrAl_2_O_4_ can be depicted as green in the CIE color diagram. The insets in Figure 5a illustrate the photographs of the undoped SrAl_2_O_4_ when the irradiation of the ultraviolet laser is turned on (left) and off (right). The inset at the right side of Figure 5a verifies the green color of the afterglow of undoped SrAl_2_O_4_. The green afterglow of undoped SrAl_2_O_4_ is visible to the human eye in the dark for about 10 s. Figure 5b depicts the afterglow decay profile of undoped SrAl_2_O_4_. This afterglow decay profile was taken after the phosphors were exposed to the 325 nm irradiation of the ultraviolet laser for 5 min. As can be seen in Figure 5b, it takes about 60 s for the luminance of the afterglow to reach the threshold value of 0.32 mcd/m^2^. Insets in Figure 5b depict the afterglow photographs of the undoped SrAl_2_O_4_ taken at 0, 5, and 10 s after the extinction of the irradiation of the ultraviolet laser. Figure 5c shows the afterglow spectra of undoped SrAl_2_O_4_ measured at different delays times after the removal of the ultraviolet illumination of the He-Cd laser (325 nm, 13 mW). These afterglow spectra were acquired with the PL spectrophotometer coupled with a CCD camera. As shown in Figure 5c, the profile of the afterglow spectrum does not change over time, but the peak intensity of the afterglow spectrum decreases with the time. The longer the time is delayed, the lower the peak intensity is of the afterglow spectrum. Additionally, no obvious shift in the peak position of the afterglow spectra can be detected when the delay time increases from 1 to 6 s.

The green afterglow of undoped SrAl_2_O_4_ is quite similar to those of Eu^2+^ singly doped SrAl_2_O_4_ [32] and Eu^2+^ and Dy^3+^ codoped SrAl_2_O_4_ [1,2,5,6]. It is worth noting that the undoped SrAl_2_O_4_ in this work is free of Eu^2+^ contamination. Consequently, the green afterglow of undoped SrAl_2_O_4_ highlights the possibility of intrinsic defects in SrAl_2_O_4_, acting as the luminescence center of green afterglow.

### 3.5. TL Glow Curve of Undoped SrAl_2_O_4_

**Figure 6:**
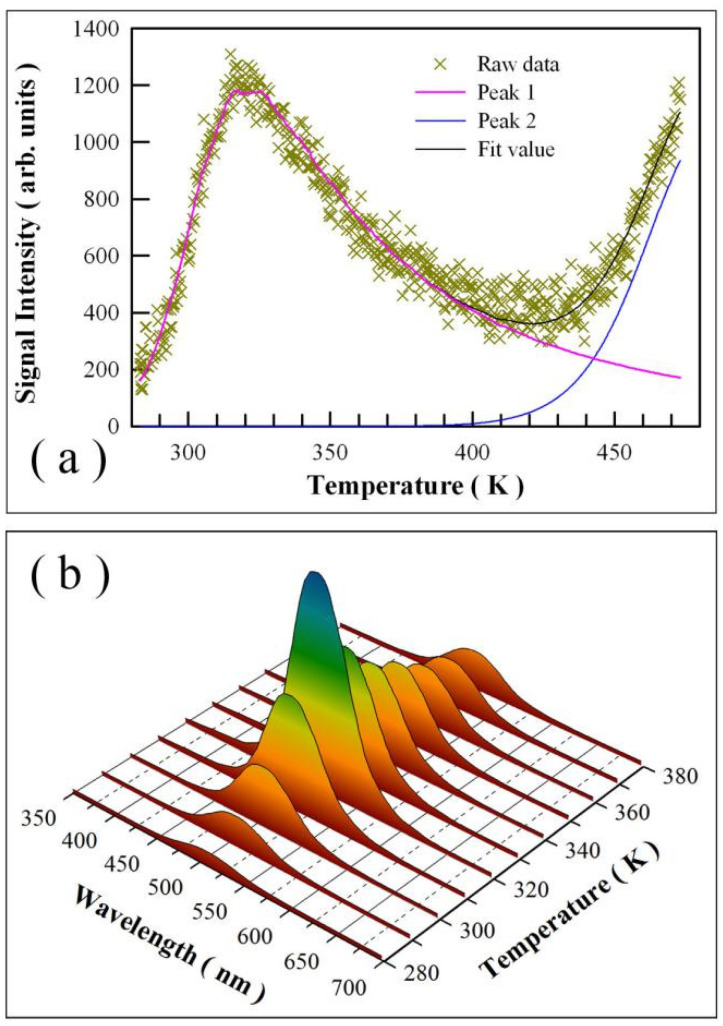
(**a**) Thermoluminescence glow curve of undoped SrAl_2_O_4_ and its deconvolution with general order kinetics. The temperature rising rate was 2 K/min. (**b**) Thermoluminescence emission spectrum of undoped SrAl_2_O_4_ measured at different temperatures.

The activation energies (i.e., trap depths) of trapping levels in undoped SrAl_2_O_4_ can be determined by analyzing its TL glow curve. Figure 6 represents the TL glow curve of undoped SrAl_2_O_4_ in the temperature range of 283–473 K. The temperature rising rate is 2 K/min. Obviously, one broad band appears in the temperature range of 283–400 K, while the other band is present at high temperatures (>430 K). As can be seen in Figure 6, the TL glow curve in the range of 283–400 K is broad and asymmetric in profile with a peak at around 321 K. The half width temperatures at the low and high temperature sides are 301 and 369 K, respectively, which yields the geometry factor of the TL glow curve of 0.70. Apparently, this value of geometry factor is much larger than that of the second order kinetics (0.52). Thus, the broad TL glow curve suggests a continuous distribution of trap levels in undoped SrAl_2_O_4_, rather than a few discrete trap levels. Interestingly, Nazarov et al. reported oxygen defects related TL peak at 325 K for Eu^2+^ doped SrAl_2_O_4_ (1 mol%) [39]. To further estimate the trap depths in undoped SrAl_2_O_4_, a deconvolution of the TL glow curve with general order kinetics is carried out using a computer program given by Chung et al. [45]. Unfortunately, it is found that this TL glow curve cannot be described satisfactorily by using the general order kinetics to model the traps in undoped SrAl_2_O_4_. Based on the TL peak temperature, one can use Urbach’s formula to roughly estimate the trap depth for undoped SrAl_2_O_4_. The trap depth estimated in this way is 0.642 eV for undoped SrAl_2_O_4_. Figure 6b illustrates the TL emission spectrum of undoped SrAl_2_O_4_ measured at different temperatures. As shown in Figure 6b, the TL emission spectrum gains its intensity as the temperature increases from 280 to 320 K. Further increase in the temperature renders the TL emission spectrum to lose its intensity.

The afterglow lifetime of an afterglow material is sensitively influenced by the activation energy of a trap. Previous studies show that the ideal trap depth of a trap should be in the range of 0.4–1.0 eV [1,2,12]. If a trap is shallower than 0.4 eV, electrons in this trap will be easily depopulated at room temperature, which prevents experimental observation of afterglow from an afterglow material. As a contrast, a trap that is deeper than 1.0 eV will not allow electrons to be released at room temperature. An ideal trap is assumed to be about 0.65 eV because it is deep enough to trap an electron effectively but not too deep to prevent slow release at room temperature [1,2,18]. Obviously, the trap depth of the trap in undoped SrAl_2_O_4_ is close to the ideal value, but why is the afterglow of undoped SrAl_2_O_4_ still short-lived? It is noticed that the intensity of the TL glow curve of undoped SrAl_2_O_4_ is about two orders of magnitude lower than that of Eu^2+^ doped SrAl_2_O_4_ (1 mol%). The low TL intensity indicates very low trap concentration in undoped SrAl_2_O_4_. Consequently, the low population density of electron traps in undoped SrAl_2_O_4_ is one of the reasons why undoped SrAl_2_O_4_ has a short-lived afterglow.

### 3.6. Band Structures and Densities of States of Defect-Rich SrAl_2_O_4_

**Figure 7:**
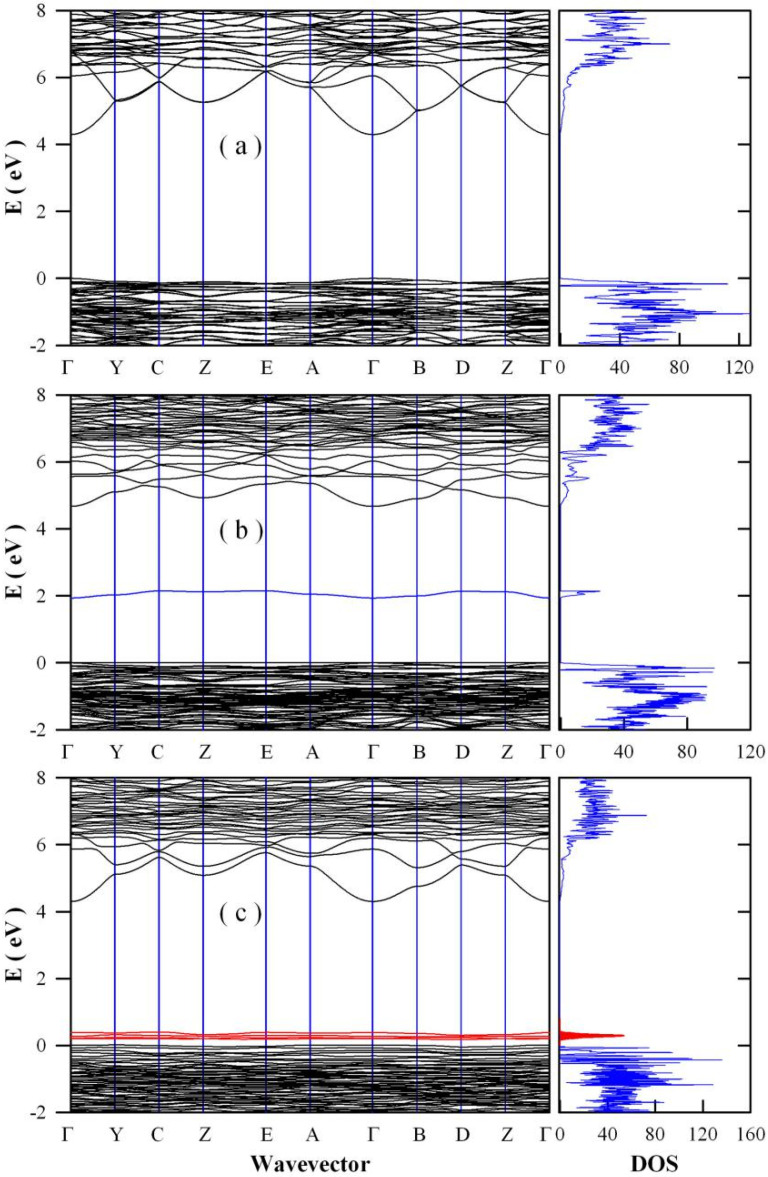
DFT calculated band structures and densities of states of SrAl_2_O_4_: (**a**) defect-free SrAl_2_O_4_; (**b**) oxygen deficient SrAl_2_O_4_ (i.e., SrAl_2_O_3.875_); and (**c**) strontium-deficient SrAl_2_O_4_ (i.e., Sr_0.875_Al_2_O_4_). The exchange–correlation functional was treated within the GGA scheme by the Perdew–Burke–Ernzerhof potential.

Oxygen and strontium vacancies are two kinds of common point defects in SrAl_2_O_4_, and they tend to generate midgap states in the bandgap of SrAl_2_O_4_. In the Supporting Materials, the first Brillouin zone of the monoclinic lattice of SrAl_2_O_4_ is given as a separate figure (Figure S1). Calculation of the band structure of CaAl_2_O_4_ crystal is performed for the high-symmetry points Г, B, D, Z, C, Y, A, E, and along the lines between them in the Brillouin zone (Figure S1). Figure 7a represents the DFT calculated band structures and densities of states of defect-free SrAl_2_O_4_. In the DFT calculations, the exchange–correlation functional was treated within the GGA scheme by the PBE potential. It can be seen in Figure 7a that the bandgap of defect-free SrAl_2_O_4_ is calculated to be 4.29 eV. Additionally, the plot of densities of states shows clearly that the bandgap of SrAl_2_O_4_ is free of any midgap states. As documented in the literature, Liu et al. and Nazarov et al. calculated the band structures and the densities of states of monoclinic SrAl_2_O_4_ by treating the exchange–correlation functional within the GGA scheme, and their bandgap values were reported to be 4.49 and 4.52 eV, respectively [46,47]. Clearly, our calculated bandgap value (4.29 eV) is consistent with those reported data. However, these calculated bandgap values are significantly underestimated when compared to the experimental bandgap value of SrAl_2_O_4_ (6.52 eV) [32,33,34,35]. To overcome the problem of bandgap underestimation, scissors operator is implemented to correct the significantly underestimated bandgap by shifting the conduction bands upwards so that the bandgap of defect-free SrAl_2_O_4_ is adjusted to be 6.52 eV. After scissors operation, the calculated band structures and densities of states of defect-free SrAl_2_O_4_ are shown in Figure S2a.

The cut-off energy for the plane waves was 75 Hartree, which equals to 2040.75 eV and seems very large. In our case, the cut-off energy of 75 Hartree is applied to the entire assembly of the 56 atoms (i.e., 8 Sr atoms, 16 Al atoms and 32 O atoms) in the 1 × 1 × 2 supercell. On one hand, setting the value of cut-off energy depends on the number of atoms in the cell. The more atoms there are in the cell, the higher the cut-off energy is for the cell. As documented in the literature, the cut-off energy of 500 eV (about 18.4 Hartree) is often chosen for a unit cell containing several atoms in the density functional calculations. Consequently, a much higher cut-off energy should be chosen for the supercell that contains 56 atoms. On the other hand, setting the value of cut-off energy depends on the accuracy of the calculation. A higher cut-off energy should be chosen for a higher accuracy. It is found that the total energy of the supercell converges to an accuracy better than 0.01 mV/atom when the cutoff energy is 75 Hartree. Additionally, the self-consistent calculation converges to an accuracy not worse than 0.2 mV/atom when the cutoff energy is 20 Hartree. In order to have an accuracy better than 0.01 mV/atom, we have set 75 Hartree as the cut-off energy for the density functional calculations in this work.

Figure 7b depicts the DFT calculated band structures and densities of states of oxygen-deficient SrAl_2_O_4_ (i.e., SrAl_2_O_3.785_). As shown in Figure 7b, the calculated bandgap of the oxygen deficient SrAl_2_O_4_ is 4.67 eV, and oxygen vacancy introduces midgap states in the bandgap of SrAl_2_O_4_. Peaking at E_V_ + 2.04 eV, these defect energy levels are distributed between E_V_ + 1.94 and E_V_ + 2.15 eV. Using the scissors operator to overcome the problem of bandgap underestimation, the bandgap of oxygen-deficient SrAl_2_O_4_ is adjusted to be 6.52 eV, in the meanwhile the oxygen vacancy introduced defect energy levels are distributed from E_V_ + 2.69 eV to E_V_ + 2.99 eV. After scissors operation by multiplying the defect and conduction energy levels with a scale factor of 1.396, the calculated band structures and densities of states of oxygen-deficient SrAl_2_O_4_ are shown in Figure S2b. These defect energy levels can be clearly identified in the plot of density of states, which exhibits a prominent peak at E_V_ + 2.84 eV. Employed DFT with a hybrid exchange and correlation functional, Finley et al. reported that oxygen vacancies form favorably at energies more than 2 eV above the valence band of SrAl_2_O_4_ [12]. At this point, the energy level of oxygen vacancy derived from our density functional calculation (E_V_ + 2.84 eV) is consistent with that reported by Finley et al.

Figure 7c illustrates the DFT calculated band structures and densities of states of strontium-deficient SrAl_2_O_4_ (i.e., Sr_0.875_Al_2_O_4_). As shown in Figure 7c, the calculated bandgap of strontium-deficient SrAl_2_O_4_ is 4.30 eV. The defect energy levels generated by strontium vacancy are distributed between E_V_ + 0.10 eV and E_V_ + 0.40 eV. Clearly, the defect energy levels of strontium-deficient SrAl_2_O_4_ are distributed closely to the top of the valence band of SrAl_2_O_4_. From the plot of density of states, we can see that the peak of the density of states of these defect energy levels is located at E_V_ + 0.30 eV. After the scissors operation to overcome the bandgap overestimation problem, the bandgap of strontium-deficient SrAl_2_O_4_ is adjusted to 6.52 eV whilst the defect energy levels generated by strontium vacancy are distributed between E_V_ + 0.15 and E_V_ + 0.61 eV. In the density of states plot, the peak of the density of states of these defect energy levels is adjusted to E_V_ + 0.45 eV. After scissors operation by multiplying the defect and conduction energy levels with a scale factor of 1.516, the calculated band structures and densities of states of strontium-deficient SrAl_2_O_4_ are shown in Figure S2c. Employed DFT with a hybrid exchange and correlation functional, Finley et al. reported that strontium vacancies form favorably at energies less than 1 eV above the valence band [12]. At this point, our calculated energy level of strontium vacancy (E_V_ + 0.45 eV) agrees well with that reported by Finley et al.

These intrinsic defects in SrAl_2_O_4_ are well known to work as luminescence centers of PL [15,16]. Moreover, they can also work as carrier traps. Being positively charged, oxygen vacancy can work as an electron trap because it attracts electron around it. Similarly, strontium vacancy can work as a hole trap because it is negatively charged [5]. Therefore, these intrinsic defects will play important roles in the PL and afterglow of undoped SrAl_2_O_4_.

### 3.7. EPR Spectrum of Undoped SrAl_2_O_4_

**Figure 8:**
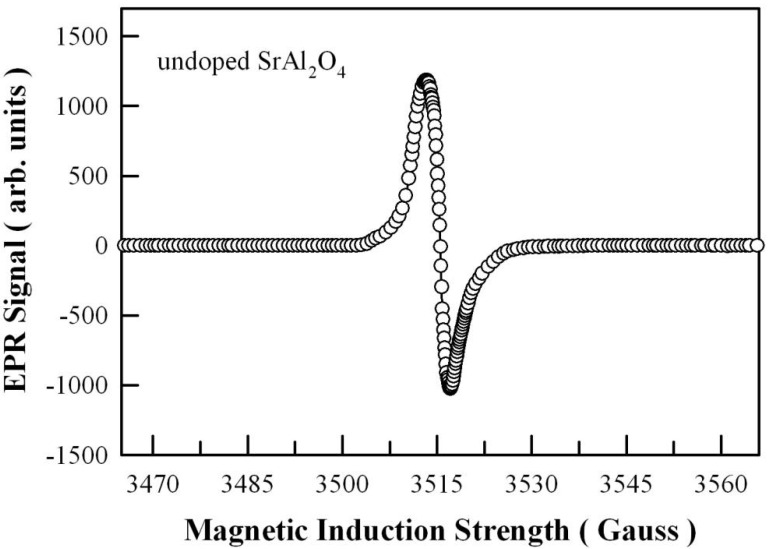
EPR spectrum of undoped SrAl_2_O_4_ measured at room temperature. Microwave frequency: 9.856 GHz; microwave power: 20 mW.

The presence of oxygen vacancies in undoped SrAl_2_O_4_ can be evidenced by EPR spectroscopy, which provides information on chemical species with unpaired electrons. Figure 8 illustrates the EPR spectrum of undoped SrAl_2_O_4_ measured at room temperature. The microwave frequency is 9.856 GHz. From its crossover point, the center field of this resonance is determined to be 3515.5 G, and then the gyromagnetic g value of the signal is determined to be 2.0032. It is known that the g value of free electron is 2.0023. Apparently, the g value derived in this work for undoped SrAl_2_O_4_ is close to that of free electrons. Holsa et al. pointed out that even undoped CaAl_2_O_4_ contains trapped electrons, probably in oxygen vacancies [48]. Our recent studies showed that trapped electrons are present in undoped SrSO_4_ and undoped CaAl_2_O_4_ [15,18]. Consequently, Figure 8 demonstrates that unpaired electrons are present in undoped SrAl_2_O_4_. Moreover, these unpaired electrons probably originate from electrons trapped in positively charged oxygen vacancies.

Because F^+^ center contains unpaired electrons, the concentration of F^+^ center in undoped SrAl_2_O_4_ can be quantitatively determined by doubly integrating the first derivative EPR spectrum. After subtraction of a background EPR signal, the concentration of F^+^ centers within undoped SrAl_2_O_4_ can be determined by comparing the double integration of the EPR signal to that of a reference standard (MnO powder). The number of spins in the reference standard is 3.34 × 10^19^ spins/g. The spin density is determined to be around 3.0 × 10^16^ spins/g for undoped SrAl_2_O_4_. Thus, the concentration of F^+^ center in undoped SrAl_2_O_4_ is around 8.4 × 10^15^ cm^−3^. However, the concentrations of F or F^2+^ centers in SrAl_2_O_4_ cannot be determined with the EPR technique, since neither F nor F^2+^ has an unpaired electron. Thus, the study of the concentration of charged states of oxygen vacancies in SrAl_2_O_4_ demands dedicated research efforts through experiments.

### 3.8. Intrinsic Defects Related PL and Afterglow Mechanisms for SrAl_2_O_4_

**Figure 9:**
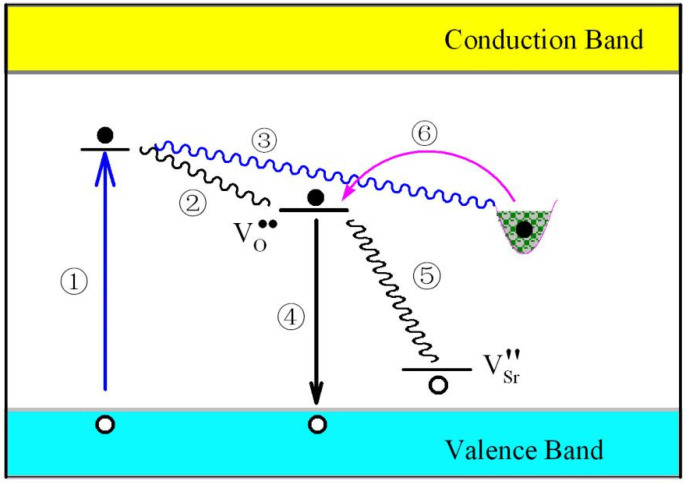
Schematic illustration of the PL and afterglow mechanisms of undoped SrAl_2_O_4_. Process ①: absorption of the excitation energy by intrinsic defects in SrAl_2_O_4_. Process ②: non-radiative relaxation of hot electrons to oxygen vacancies in SrAl_2_O_4_. Process ③: non-radiative relaxation of hot electrons to electron traps (oxygen vacancies) in SrAl_2_O_4_. Process ④: radiative recombination of electrons captured at oxygen vacancy with holes in the valence band of SrAl_2_O_4_, resulting in a broad PL band. Process ⑤: radiative recombination of electrons captured at oxygen vacancies with holes captured at strontium vacancies, yielding another PL band. Process ⑥: thermal release of electrons from electron traps in SrSO_4_, followed by radiative recombination via process ⑤ to yield the green afterglow.

The intrinsic defects related PL and afterglow mechanisms are displayed in Figure 9 for undoped SrAl_2_O_4_. As sketched in Figure 9, the midgap states of oxygen and strontium vacancies are located at E_V_ + 2.84 eV and E_V_ + 0.45 eV, respectively. An electron trap is presented in the middle of Figure 9 to show the possible role played by oxygen vacancy in undoped SrAl_2_O_4_. Upon the photoexcitation of the ultraviolet light at 325 nm (3.82 eV), a fraction of excitation energy is absorbed by SrAl_2_O_4_ lattice since practical SrAl_2_O_4_ contains various kinds of intrinsic defects (process ①). Non-radiative relaxations take place when hot electrons are captured by the oxygen vacancies (process ②) or by the electron traps (process ③) present in SrAl_2_O_4_. According to the mechanisms proposed in Figure 9, three different paths become available for the subsequent radiative recombinations: (i) the electrons captured at oxygen vacancy recombine radiatively with holes in the valence band of SrAl_2_O_4_, resulting in a broad PL band with its peak energy determined by the energy level of the oxygen vacancy in the bandgap of SrAl_2_O_4_ (process ④); (ii) the electrons captured at oxygen vacancy recombine radiatively with holes trapped at the strontium vacancy, yielding another broad PL band with its peak energy determined by the energy difference between the oxygen and strontium vacancies in the bandgap of SrAl_2_O_4_ (process ⑤); and (iii) upon thermal stimulation and/or photon stimulation, the electrons are released from electron traps (process ⑥) to recombine radiatively with holes trapped at the strontium vacancy (process ⑤). The third path is a combined process that is coupled with the processes ⑥ and ⑤. This combined process results in a luminescence band which is identical to that of the second path.

When the photoexcitation is stopped, processes ①–④ are terminated immediately, but processes ⑥ and ⑤ are kept working. Under thermal activation, electrons trapped at the electron traps can be released (process ⑥), and an afterglow can be resulted if the detrapped electrons recombine radiatively with holes trapped at the strontium vacancy (process ⑤). Hence, according to Figure 9, green afterglow can be expected for undoped SrAl_2_O_4_. Clearly, this afterglow mechanism involves the gradual release of electrons from electron traps (i.e., oxygen vacancies) followed by electron migration to strontium vacancies. Consequently, the couple of oxygen and strontium vacancies can be ascribed to be the luminescence center of the green afterglow of undoped SrAl_2_O_4_.

If the proposed mechanisms in Figure 9 are reasonable, we can expect that: (i) the PL spectrum of undoped SrAl_2_O_4_ should consist of two subbands; (ii) one PL subband should be peaked at about 2.39 eV (518 nm) while the other PL subband should be peaked at about 2.84 eV (436 nm); and (iii) the afterglow of undoped SrAl_2_O_4_ should be peaked at 2.39 eV (518 nm). Qualitatively speaking, the record of two subbands in the PL spectrum of undoped SrAl_2_O_4_ (in Figure 4) and the observation of green afterglow peaking at 2.38 eV (520 nm) (in Figure 5) have matched well with the expectations. Quantitatively speaking, the peak energies of the two PL subbands, namely 2.63 eV (471.6 nm) for the first subband and 3.13 eV (396.4 nm) for the second subband in Figure 4, have fallen far short of our expectations (i.e., 2.39 eV for the first subband and 2.84 eV for the second subband). The maximum difference between the experimental peaking energy and the expected one is 0.29 eV. Two factors might be responsible for the discrepancies. The first factor is that the inherent limitation of the DFT calculations makes it difficult to exactly determine the defect energy levels for solids. The second factor is that the weak PL subband peaking at about 2.63 eV generates significant error in the PL deconvolution. 

Once the solid is synthesized, the energy levels of oxygen and calcium vacancies in the bandgap of SrAl_2_O_4_ are stable over time if the crystal structure of defect-containing SrAl_2_O_4_ does not evolve with time. Generally speaking, changes of the energy levels in the bandgap of SrAl_2_O_4_ take place only when the crystal structure of defect-containing SrAl_2_O_4_ is modified by subsequently treatments, such as thermal annealing at elevated temperatures and high-energy particle irradiation. As a contrast to the stable energy levels, the population of intrinsic defects is strongly affected by the synthesis method and subsequent treatments of the material. The stability of the energy levels of these defects in SrAl_2_O_4_ over time has been evidenced by the unchanged profile of the steady-state PL spectrum, even after storage of the sample at room temperature for a couple of years. In spite of the good stability of the defect energy levels, any electron which exists in the energy levels is in a meta-stable state and will decay with time via trap levels or defect energy levels in the band gap. The decay of electron via the trap is evidenced by the afterglow decay curve shown in Figure 5b. For the electrons in the energy levels of oxygen vacancy, they depopulate over time, and the decay of electron via the energy levels of oxygen and strontium vacancies is discussed in the following section.

### 3.9. PL Decay Curves of Undoped SrAl_2_O_4_

**Figure 10:**
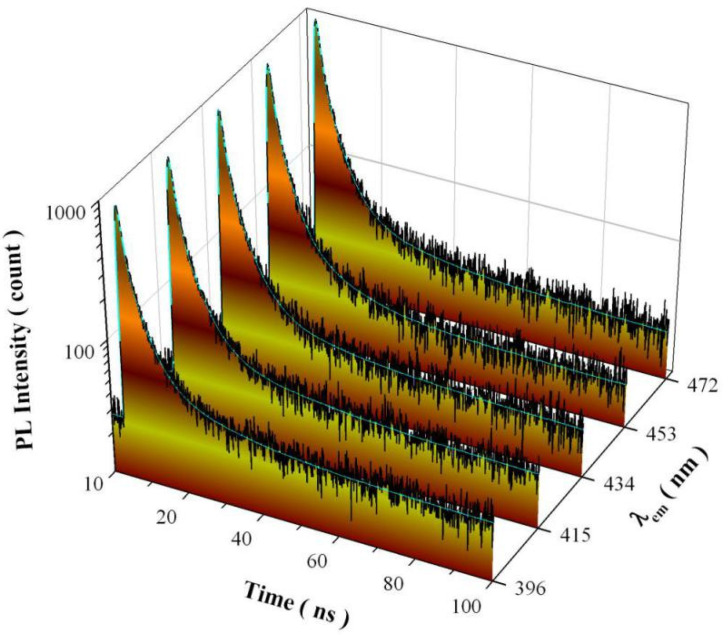
PL decay curves (black) and exponential reconvolution fits (cyan) of undoped SrAl_2_O_4_ at different detection wavelengths. The excitation was the 320 nm pulsed light with a pulse period of 100 ns.

As discussed above, undoped SrAl_2_O_4_ possesses three distinctly different paths for radiative recombination, which are namely described by process ④, process ⑤, and the combined process of ⑥ with ⑤. If so, there should be three decay components in the PL decay curve of undoped SrAl_2_O_4_. Figure 10 shows the PL decay curves (black) and exponential reconvolution fits (cyan) of undoped SrAl_2_O_4_ at detection wavelengths of 396, 415, 434, 453, and 472 nm, respectively. The excitation is the 320 nm pulsed light with a pulse period of 100 ns. Instrument response function is measured for each PL decay curve. Our exponential reconvolution analysis reveals that each PL decay curve is best described by a tri-exponential decay model. Exponential reconvolution fitting parameters of the PL decay curves are summarized in Table 1 for undoped SrAl_2_O_4_. As can be seen in Table 1, there are three exponential decay components in each PL decay curve. For example, the time constants of the three exponential decays are 0.914, 3.705, and 18.335 ns for the PL decay of undoped SrAl_2_O_4_ measured at the detection wavelength of 396 nm. As described previously, the average lifetime can be calculated with parameters of the pre-exponential factors and time constants [18,21]. The averaged lifetime is calculated to be 6.49 ns for the PL decay curve of undoped SrAl_2_O_4_ measured at the detection wavelength of 396 nm. As the detection wavelength increases from 396 to 472 nm, the averaged lifetime of the PL decay curve varies in the range of 4.65–6.49 ns. Thus, the data in Figure 10 confirm the presence of three distinctly different recombination channels for the radiative recombinations in undoped SrAl_2_O_4_.

## 4. Summary

Undoped SrAl_2_O_4_ nanocrystals were prepared via the sol–gel combustion to investigate their PL and afterglow properties. The steady-state PL spectrum of undoped SrAl_2_O_4_ can be deconvoluted into two subbands peaking at 2.63 eV (472 nm) and 3.13 eV (396 nm), respectively. When the 325 nm irradiation of the helium–cadmium laser (13 mW) is turned off, the green afterglow from undoped SrAl_2_O_4_ is visible to the human eye, and it takes about 60 s for the luminance of the afterglow to reach the threshold value of 0.32 mcd/m^2^. The afterglow spectrum of undoped SrAl_2_O_4_ is broad in profile peaking at about 520 nm. The TL glow curve of undoped SrAl_2_O_4_ exhibits a broad profile with a peak at about 321 K. The trap depth is estimated to be 0.642 eV using Urbach’s formula. On the basis of density functional calculations for oxygen-deficient SrAl_2_O_4_ and strontium-deficient SrAl_2_O_4_, the midgap states introduced by oxygen and strontium vacancies are located at 2.84 eV and 0.45 eV above the valence band of SrAl_2_O_4_, respectively. Our data have demonstrated that both the PL and the afterglow of undoped SrAl_2_O_4_ are correlated with the oxygen and strontium vacancies in SrAl_2_O_4_. These findings help in exploring the afterglow mechanisms of SrAl_2_O_4_-based afterglow materials.

## Figures and Tables

**Table 1:** Exponential reconvolution fitting parameters of the PL decay curves of undoped SrAl_2_O_4_ at different emission wavelengths (λ_em_). The excitation is the 320 nm pulsed light with a pulse period of 100 ns.

λ_em_ (nm)	*A*_0_	*I*_1_	*τ*_1_ (ns)	*I*_2_	*τ*_2_ (ns)	*I*_3_	*τ*_3_ (ns)	τ_avg_ (ns)	χ^2^
396	27.825	0.130	0.9140	0.062	3.7050	0.006	18.3353	6.49	0.982
415	25.736	0.125	1.0759	0.054	4.2216	0.003	22.6141	6.14	0.969
434	22.908	0.135	1.0909	0.056	4.3042	0.002	23.3102	5.25	1.026
453	21.369	0.109	0.7717	0.076	3.0503	0.008	11.9451	4.65	0.939
472	22.942	0.120	1.0084	0.062	3.9454	0.004	19.6329	5.92	1.033

## Data Availability

The data presented in this study are available on request from the corresponding author.

## Supplementary Materials

The following are available online at https://www.mdpi.com/article/10.3390/nano11092331/s1, Figure S1: The first Brillouin zone for the monoclinic lattice of SrAl_2_O_4_. Figure S2: DFT calculated band structures and densities of states of SrAl_2_O_4_ after scissors operation: (a) defect-free SrAl_2_O_4_; (b) oxygen deficient SrAl_2_O_4_ (i.e., SrAl_2_O_3.875_); and (c) strontium-deficient SrAl_2_O_4_ (i.e., Sr_0.875_Al_2_O_4_). The exchange–correlation functional was treated within the GGA scheme by the Perdew–Burke–Ernzerhof potential.
