# Supplementary material for: Green Afterglow of Undoped SrAl2O4
Source: Nanomaterials (Basel). 2021 Sep 9;11(9):2331. doi: 10.3390/nano11092331 (PMC8467732; doi:10.3390/nano11092331)
Supplement: Supplementary file 1 [file nanomaterials-11-02331-s001.zip › nanomaterials-1350515-supplementary.pdf]

Supporting Materials: Green Afterglow of Undoped $SrAl_2O_4$

Bao–gai Zhai and Yuan Ming Huang*

School of Microelectronics and Control Engineering, Changzhou University, Changzhou 213164, China

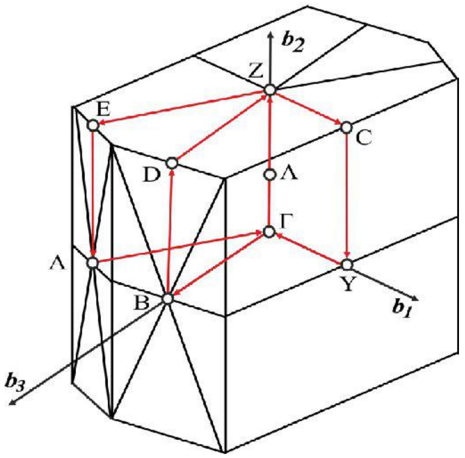

Figure S1. The first Brillouin zone for the monoclinic lattice of $SrAl_2O_4$.

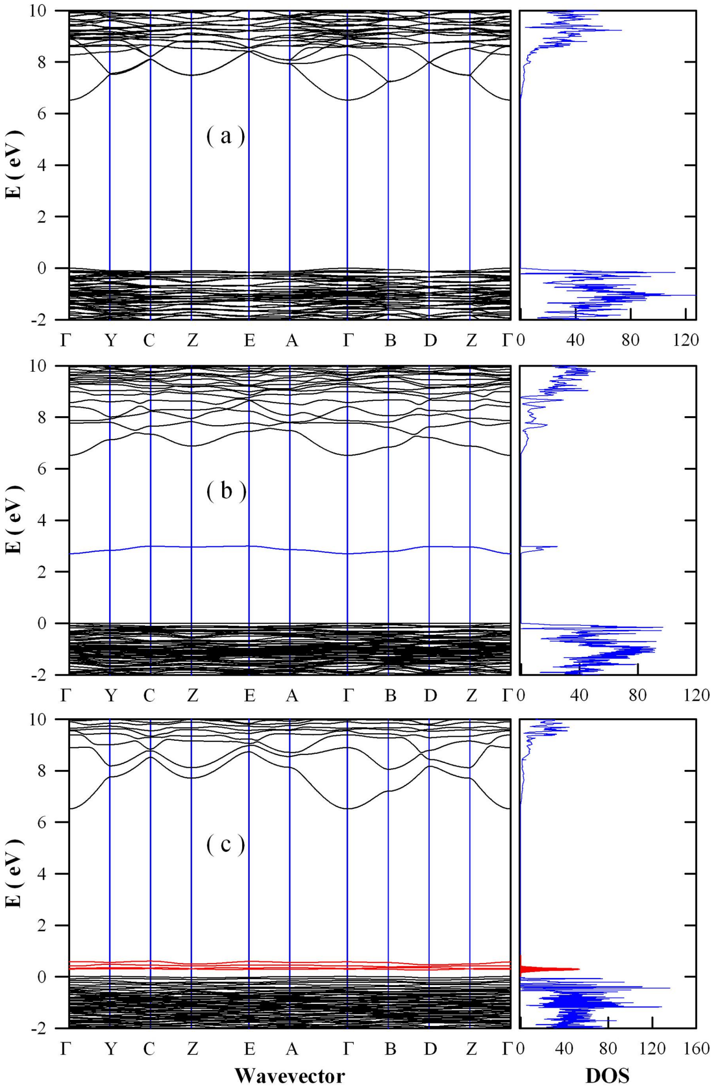

Figure S2. DFT calculated band structures and densities of states of $SrAl_2O_4$ after scissors operation: (a) defect–free $SrAl_2O_4$; (b) oxygen deficient $SrAl_2O_4$ (i.e., $SrAl_2O_{3.875}$); and (c) strontium–deficient $SrAl_2O_4$ (i.e., $Sr_{0.875}Al_2O_4$). The exchange–correlation functional was treated within the GGA scheme by the Perdew–Burke–Ernzerhof potential.
